# Household reporting of childhood respiratory health and air pollution in rural Alaska Native communities

**DOI:** 10.3402/ijch.v73.24324

**Published:** 2014-05-09

**Authors:** Desirae N. Ware, Johnnye Lewis, Scarlett Hopkins, Bert Boyer, Luke Montrose, Curtis W. Noonan, Erin O. Semmens, Tony J. Ward

**Affiliations:** 1 Department of Biomedical & Pharmaceutical Sciences, Center for Environmental Health Sciences, University of Montana, Missoula, MT, USA; 2 Community Environmental Health Program, University of New Mexico, Albuquerque, NM, USA; 3 Center for Alaska Native Health Research, University of Alaska Fairbanks, Fairbanks, AK, USA

**Keywords:** air pollution, Alaska, asthma, children, respiratory tract infections, surveys

## Abstract

**Background:**

Air pollution is an important contributor to respiratory disease in children.

**Objective:**

To examine associations between household reporting of childhood respiratory conditions and household characteristics related to air pollution in Alaska Native communities.

**Design:**

In-home surveys were administered in 2 rural regions of Alaska. The 12-month prevalence of respiratory conditions was summarized by region and age. Odds ratios (ORs) were calculated to describe associations between respiratory health and household and air quality characteristics.

**Results:**

Household-reported respiratory health data were collected for 561 children in 328 households. In 1 region, 33.6% of children aged <5 years had a recent history of pneumonia and/or bronchitis. Children with these conditions were 2 times more likely to live in a wood-heated home, but these findings were imprecise. Resident concern with mould was associated with elevated prevalence of respiratory infections in children (ORs 1.6–2.5), while reported wheezing was associated with 1 or more smokers living in the household. Reported asthma in 1 region (7.6%) was lower than national prevalence estimates.

**Conclusions:**

Findings suggest that there may be preventable exposures, including wood smoke and mould that affect childhood respiratory disease in these rural areas. Additional research is needed to quantify particulate matter 2.5 microns in aerodynamic diameter or less and mould exposures in these communities, and to objectively evaluate childhood respiratory health.

Exposure to air pollution in both ambient and indoor environments is known to contribute to a variety of respiratory health outcomes (1–4), including a 2- to 3-fold increased risk of acute respiratory tract infections and increased asthma morbidity in children (5–9). Worldwide, acute respiratory infections are the most common cause of childhood illness and a leading cause of mortality (10–12). Although acute respiratory infection in the United States is not a leading cause of mortality, lower respiratory tract infection (LRTI) remains an important cause of childhood morbidity. Bronchitis, bronchiolitis and pneumonia are the most common forms of LRTI among US children, and disabling infections can last from days to weeks. LRTI accounts for 27% of hospitalizations among children aged <5 years, and LRTI outpatient visits in this age group occur annually in 133 per 1,000 children (13).

American Indian and Alaska Native (AI/AN) children experience the highest burden of LRTI in the US (13, 14), with infant and child hospitalizations influenced disproportionally by LRTIs and infectious diseases (15–17). In the Yukon-Kuskokwim region of Alaska, hospitalization visits for respiratory syncytial virus (RSV) in Alaska Native infants (aged <1 year) have reached 5 times the national US average, and nearly 1 in 5 Alaska Native infants are hospitalized each year for LRTI (18, 19). In the Alaska Indian Health Service (IHS) region, hospitalization and outpatient visits for LRTI are 2- to 3-fold greater than in other US children (13), and overall hospitalization visits for bronchiolitis and infectious disease are higher compared to all other IHS regions (14, 15, 17).

Asthma is the most common chronic childhood illness, affecting more than 7 million children in the US (20). For more than a decade, the annual prevalence of asthma has increased by an average of 1.2%, resulting in over 450,000 hospitalizations, nearly 2 million emergency department visits and 10.5 million missed days of school each year (21). Asthma has also been shown to disproportionately affect certain age groups, socio-economic classes and minority groups including children, low-income and African American populations. However, little is known about the prevalence of asthma among Alaska Native children, as they have historically been underrepresented in the paediatric asthma literature. Existing studies describing asthma among AI/AN children have largely relied on the review of existing medical records or the use of aggregated data, with the reported results highly variable (22–26).

The study described herein was conducted as part of a project that identified regional and community specific air pollution issues of concern (27) and characterized respiratory health (LRTI and asthma) in children at the community level through door-to-door surveys. In this manuscript, we examine associations between household reporting of childhood respiratory conditions and household characteristics related to indoor and ambient air pollution in 2 distinct, rural areas of Alaska.

## Methods

### Study regions

Between September 2011 and March 2012, questionnaires were administered within 7 rural Alaska Native communities located in 2 regions of Alaska. Three communities were located in the Yukon-Kuskokwim region (Region 1) and 4 communities were located in the Ahtna region (Region 2). Additional description of these regions is provided in Ware et al. (27).

### Participants and study design

Within each of these 7 rural communities, demographics, household reporting on air quality sources and perceptions, and history of childhood respiratory health symptoms and conditions were collected through 2 community-based household questionnaires. Eligibility criteria for participation included being (a) a current household member, (b) aged over 18 years and (c) a community resident for at least 8 of the previous 12 months. The first questionnaire (hereafter referred to as “air quality questionnaire”) focused on household characteristics, identifying sources of particulate matter 2.5 microns (PM_2.5_) in aerodynamic diameter or less in both the indoor and ambient environment, and individual concerns with air quality. Results from this questionnaire have been previously reported in Ware et al. (27).

The second instrument (hereafter referred to as “respiratory health questionnaire”) focused on characterizing respiratory infections and conditions among children aged <18 years living in the 7 rural communities. Children were not recruited as direct participants, but data on their health were captured through questionnaires completed by a household representative. Respiratory health questionnaires were based on the International Study of Asthma and Allergies in Childhood questionnaire and focused on LRTI (bronchitis and pneumonia), asthma and asthma-related symptoms (28). Parents were additionally asked to report on specific respiratory conditions including the “flu,” cold, and throat and ear infections. These instruments were completed once per household (1 survey per child resident) and asked questions about respiratory health during the preceding 12 months (i.e. “In the last 12 months, has your child had any of the following conditions?”). Only households with children meeting age eligibility criteria were invited to participate in this portion of the study. Each questionnaire participant (1 per household) was informed of the research and provided signed consent prior to participating in the study. In Region 1, questionnaires were translated into Yup'ik for non-English speakers, and a translator was also available during the questionnaire process to translate the consent and questionnaires. In Region 2, English versions of the survey were exclusively used.

### Data analysis

The prevalence of respiratory conditions (cold, flu, bronchitis and pneumonia) and symptoms in the 12 months preceding the questionnaire were summarized by region. Differences by region were evaluated using chi-square tests, or Fisher's exact test when necessary. Reported child respiratory infections were evaluated separately for children aged <2 years, aged <5 years and aged 5–18 years. For Region 1 only, reporting of child health outcomes was additionally evaluated with respect to household characteristics. Note that this evaluation was not conducted for Region 2 because of the lower number of participants in that region.

To evaluate these associations and to account for multiple children within homes, we used generalized estimating equations (GEE) with a logit link to estimate odds ratios (ORs) and 95% confidence intervals (95% CIs), assuming an exchangeable correlation structure. Adults responding to questions about specific respiratory conditions or symptoms of given household children were allowed to respond “I don't know” (≤1.6% for conditions and ≤3.6% for asthma symptoms). For comparisons, we treated this response as “no.” Questions with no recorded response (≤0.7%) were considered missing. All data were analysed using SAS v9.3 software (Cary, NC).

### Human subjects review

This study was approved by the University of Montana Institutional Review Board, the Alaska Area Institutional Review Board and the Yukon-Kuskokwim Health Corporation's Executive Board of Directors. Tribal village council approval was also received from each participating community prior to conducting activities related to this study.

## Results

The 7 communities that participated in this study ranged in size from approximately 100 to 700 people. In total, questionnaires were conducted within 328 households, with 241 households in Region 1 and 87 households in Region 2 participating. Respiratory health data were collected for 561 children within these homes (475 children in Region 1 and 86 children in Region 2). Of the 561 children, 53.6% were male and 157 were aged <5 years. Based on 2010 Census data and community household lists, surveys were conducted in approximately 70% of households in both regions, and respiratory health data were captured for approximately 85% of children in Region 1. Because available Census data for Region 2 also included children in areas outside the confines of tribal communities where participation was solicited for this study, we were unable to accurately estimate the proportion of children captured by the Region 2 questionnaires.

Table I summarizes the reporting of prevalent respiratory conditions (flu, cold, throat and ear infection, bronchitis and pneumonia) in the previous 12 months among all children (children aged <5 years and 5 to 17 years). For the selected respiratory conditions, additional analyses were conducted for younger populations, but these analyses were not informative because of the small sample size. For example, in Region 1, we captured parental responses for only 65 children aged <2 years (13.7%) and 31 children aged <1 year (6.5%). For both age groups (aged <5 years and 5 to17 years), the frequencies of reported flu, cold, and throat and ear infections were similar between the 2 regions. In Region 1, nearly 50% of children aged <5 years were reported to have experienced the flu compared to 35% in Region 2 (p=0.28). In both regions, approximately 70% of children aged 5 to 17 years and 80% aged <5 years had a cold, whereas 30% of children aged <5 years in Region 2 and 23% in Region 1 had a throat infection in the past year (p=0.57). As expected, reported ear infection was more common among children aged <5 years, with the frequencies of reported ear infection similar between the two regions in this age group (48.5% and 45.0%, p=0.77).

**Table I:** Reporting of respiratory conditions in previous 12 months among all children

	Children aged<5 years					Children aged 5 to 17 years				
		
	Region 1		Region 2			Region 1		Region 2		
						
	n	Number positive (%)	n	Number positive (%)	p*	n	Number positive (%)	n	Number positive (%)	p*
Flu	136	65 (47.8)	20	7 (35.0)	0.28	337	123 (36.5)	66	21 (31.8)	0.47
Cold	136	111 (81.6)	20	16 (80.0)	0.77	337	226 (67.1)	66	49 (74.2)	0.25
Throat infection	136	31 (22.8)	20	6 (30.0)	0.57	337	141 (41.8)	66	26 (39.4)	0.71
Ear infection	136	66 (48.5)	20	9 (45.0)	0.77	337	54 (16.0)	66	6 (9.1)	0.15
Bronchitis	136	33 (24.3)	20	1 (5.0)	0.08	337	39 (11.6)	66	4 (6.1)	0.19
Pneumonia^a^	131	25 (19.1)	20	0 (0.0)	0.05	336	31 (9.2)	66	1 (1.5)	0.03
Respiratory Hospital/ED^b^	136	33 (24.3)	20	3 (15.0)	0.60	337	71 (21.1)	66	14 (21.2)	0.98

a Five responses missing for Region 1 children aged<5 years (n=131), and 1 response missing for Region 1 children aged 5 to 17 years (n=336).

b Reported for lifetime rather than previous 12 months.

* p value obtained from chi-square test or Fisher's exact test when necessary.

Overall, Region 1 respondents indicated a higher occurrence of LRTI (bronchitis and/or pneumonia) among children. For children aged 5 to 17 years, the frequency of reported bronchitis was nearly double in Region 1 compared to Region 2 (11.6% compared to 6.1%, p=0.19), and the frequency of reported pneumonia was significantly higher (9.2% compared to 1.5%, p=0.03). The regional differences in reporting of LRTI were even greater among children aged<5 years, with 33.6% of Region 1 children in this age group having bronchitis and/or pneumonia during the past year compared to 5% in Region 2 (p=0.008). Across both regions, approximately 21% of children aged 5 to 17 years reported a lifetime hospitalization due to respiratory disease. However, reported lifetime hospitalization (due to respiratory disease) among children aged <5 years tended to be different, with nearly 25% in Region 1 compared to 15% in Region 2 (p=0.60).

Table II presents the reporting of asthma and respiratory symptoms among all children (aged<18 years). Reported asthma was slightly higher among children in Region 2, with 9.3% reporting asthma compared to 7.6% in Region 1 (p=0.60). Reported asthma medication use was discrepant between the 2 regions. In Region 2, 75% of diagnosed asthmatics were currently taking medication, with only 30.6% in Region 1 (p=0.03). Among asthma-related symptoms, wheezing without the presence of a cold was 2.5 times higher among children in Region 1 (p=0.03). The discrepancy between the 2 regions was particularly notable for reported wheezing among children without reported asthma (12.4% in Region 1 compared to 2.6% in Region 2 (p=0.01)) (results not shown). Reported occurrence of dry cough at night among children was similar in the 2 regions, as was the reporting of congestion symptoms, irritated eyes and productive cough.

**Table II:** Reporting of asthma and respiratory symptoms among Alaska Native children aged<18 years by region

	Region 1		Region 2		
			
	n	Number positive (%)	n	Number positive (%)	p*
Reported asthma	471	36 (7.6)	86	8 (9.3)	0.60
Physician diagnosed^a^	36	34 (99.4)	8	7 (87.5)	0.75
Current asthma medication^a^	36	11 (30.6)	8	6 (75.0)	0.03
Wheezing	473	70 (14.8)	85	5 (5.9)	0.03
Cough at night	473	117 (24.7)	86	18 (20.9)	0.45
Stuffy or runny nose, or sinus congestion	473	184 (38.9)	86	28 (32.6)	0.26
Watery, itchy or irritated eyes	471	55 (11.7)	86	14 (16.3)	0.23
Productive cough	473	85 (18.0)	86	17 (19.8)	0.69

a Question only asked about children with reported asthma.

* p value shown for chi-square test, or Fisher's exact when necessary, comparing responses across regions.

Tables III and IV present the ORs describing the relationship between prevalent respiratory infections in the previous 12 months and household variables among Region 1 children aged <5 years and children aged 5 to 17 years, respectively. For LRTI in children aged <5 years (Table III), the highest estimated OR point estimates were for children living in homes that were heated exclusively with wood stoves relative to homes that were heated exclusively with fuel oil. These associations were imprecise, and did not exclude the null, with ORs of 2.1 (95% CI: 0.6, 7.2) and 2.0 (95% CI: 0.7, 6.3) for pneumonia and bronchitis, respectively. When household smoking was included as a covariate, associations were similar to those observed without smoking included in analysis (results not shown). For upper respiratory infections (cold, throat infection and middle-ear infection), wood stove only homes yielded elevated but non-significant ORs relative to fuel oil only homes (OR point estimates ranged from 1.7 to 1.9; Table III). The highest OR for upper respiratory infection (cold) among children aged <5 years was observed for homes with mixed wood and oil heating relative to fuel oil only (OR=4.1; 95% CI: 1.2, 14.3).

**Table III:** Odds ratios (OR, 95% CI) for respiratory infections and household variables among children aged<5 years in Region 1

	n	Pneumonia	Bronchitis	Flu	Cold	Throat infection	Middle-ear infection
Crowding							
< 2.5	84	1.0	1.0	1.0	1.0	1.0	1.0
≥ 2.5	49	1.1 (0.4, 2.9)	1.1 (0.5, 2.7)	0.8 (0.4, 1.7)	0.4 (0.2, 1.1)	1.1 (0.5, 2.7)	0.5 (0.2, 1.1)
Heating^a^							
Fuel oil only	50	1.0	1.0	1.0	1.0	1.0	1.0
Fuel oil + woodstove	53	1.1 (0.3, 3.5)	0.7 (0.3, 2.2)	1.6 (0.7, 3.7)	4.1 (1.2, 14.3)*	1.2 (0.5, 3.1)	1.2 (0.5, 2.6)
Woodstove only	25	2.1 (0.6, 7.2)	2.0 (0.7, 6.3)	1.0 (0.3, 3.1)	1.8 (0.5, 6.3)	1.9 (0.6, 6.1)	1.7 (0.7, 4.4)
Household smoker							
No	64	1.0	1.0	1.0	1.0	1.0	1.0
Yes	70	1.2 (0.5, 3.0)	1.6 (0.7, 3.7)	1.3 (0.6, 2.8)	1.4 (0.5, 3.5)	1.3 (0.6, 3.0)	0.8 (0.4, 1.6)
Age of house							
Built 1985 or later	75	1.0	1.0	1.0	1.0	1.0	1.0
Built before 1985	53	1.2 (0.4, 3.1)	0.2 (0.1, 0.6)*	1.2 (0.5, 2.6)	0.2 (0.1, 0.7)*	1.3 (0.6, 3.0)	0.6 (0.3, 1.3)
Presence of mould^b^							
No	101	1.0	1.0	1.0	1.0	1.0	1.0
Yes	35	1.3 (0.5, 3.5)	1.7 (0.6, 4.3)	2.5 (1.0, 6.1)	2.4 (0.6, 8.7)	1.8 (0.7, 4.5)	2.2 (1.0, 5.0)
Concern of outdoor smoke^b^							
No	115	1.0	1.0	1.0	1.0	1.0	1.0
Yes	21	1.8 (0.6, 5.6)	1.8 (0.6, 4.8)	1.3 (0.5, 3.6)	0.9 (0.2, 3.7)	1.4 (0.5, 4.4)	1.9 (0.7, 5.4)
Change – Ventilation/purification^b^							
No	104	1.0	1.0	1.0	1.0	1.0	1.0
Yes	32	1.9 (0.7, 5.3)	0.5 (0.1, 1.5)	1.1 (0.5, 2.7)	0.7 (0.2, 2.0)	2.2 (0.9, 5.5)	0.8 (0.3, 1.7)

n=number of children; OR=odds ratio; CI=confidence interval.

a Does not include 8 children in homes with other type of heating.

b These non-mutually exclusive categories are based on open-ended comments by participants.

* p<0.05.

**Table IV:** Odds ratios (OR, 95% CI) for respiratory infections and household variables among children aged 5 to 17 years in Region 1

	n	Pneumonia	Bronchitis	Flu	Cold	Throat infection	Middle-ear infection
Crowding							
< 2.5	178	1.0	1.0	1.0	1.0	1.0	1.0
≥ 2.5	156	1.7 (0.8, 3.5)	1.0 (0.5, 1.9)	0.8 (0.5, 1.4)	0.8 (0.4, 1.3)	1.0 (0.6, 1.7)	0.9 (0.6, 1.4)
Heating^a^							
Fuel oil only	130	1.0	1.0	1.0	1.0	1.0	1.0
Fuel oil + woodstove	131	0.8 (0.3, 1.8)	0.7 (0.3, 1.4)	1.2 (0.7, 2.2)	1.5 (0.8, 2.7)	0.9 (0.5, 1.5)	1.3 (0.8, 2.1)
Woodstove only	60	1.5 (0.6, 4.0)	1.5 (0.7, 3.3)	1.0 (0.5, 2.3)	1.0 (0.5, 2.0)	1.5 (0.8, 3.0)	1.2 (0.6, 2.3)
Household smoker							
No	180	1.0	1.0	1.0	1.0	1.0	1.0
Yes	156	1.3 (0.6, 2.6)	1.4 (0.8, 2.5)	1.8 (1.1, 3.0)*	1.2 (0.7, 1.9)	1.4 (0.9, 2.2)	1.0 (0.7, 1.6)
Age of house							
Built 1985 or later	186	1.0	1.0	1.0	1.0	1.0	1.0
Built before 1985	140	1.2 (0.6, 2.5)	0.6 (0.3, 1.1)	1.2 (0.7, 2.0)	0.9 (0.5, 1.6)	0.9 (0.6, 1.5)	0.9 (0.5, 1.3)
Presence of mould^b^							
No	234	1.0	1.0	1.0	1.0	1.0	1.0
Yes	103	1.6 (0.5, 2.3)	1.6 (0.9, 3.0)	2.0 (1.1, 3.7)*	1.7 (0.9, 3.3)	1.6 (1.0, 2.8)	1.8 (1.2, 2.7)*
Concern of outdoor smoke^b^							
No	271	1.0	1.0	1.0	1.0	1.0	1.0
Yes	66	1.5 (0.6, 3.7)	1.4 (0.7, 2.8)	1.3 (0.7, 2.4)	2.0 (0.8, 4.5)	1.0 (0.5, 1.8)	1.4 (0.7, 2.9)
Change – Ventilation/purification^b^							
No	249	1.0	1.0	1.0	1.0	1.0	1.0
Yes	88	2.6 (1.3, 5.5)*	1.5 (0.7, 2.9)	2.2 (1.2, 3.9)*	1.8 (0.9, 3.5)	1.6 (0.9, 2.7)	1.5 (0.9, 2.4)

n=number of children; OR=odds ratio; CI=confidence interval.

a Does not include 8 children in homes with other type of heating.

b These non-mutually exclusive categories are based on open-ended comments by participants.

* p<0.05.

The presence of household smoking in Region 1 was not strongly associated with any of the reported respiratory infections among children aged <5 years. When household smoking was included in analyses of home heating and respiratory infections, the associations were similar to those described above (results not shown). Living in an older home (built before 1985) was associated with lower reported prevalence of bronchitis (OR=0.2; 95% CI: 0.1, 0.6), cold (OR=0.2; 95% CI: 0.1, 0.7) and, to a lesser degree, ear infection (OR=0.6; 95% CI: 0.3, 1.3).

When Region 1 adult respondents were asked to comment on concerns regarding both the indoor and outdoor environment (as recorded by the air quality questionnaires), among the most frequently expressed concerns were indoor mould (28%) and outdoor sources of smoke (21%) (27). When investigating associations between the responses to the 2 surveys, we discovered that respiratory infections in children were more likely to be reported by adult residents who also had expressed a concern about indoor mould, notably for flu (OR=2.5; 95% CI: 1.0, 6.1), cold (OR=2.4; 95% CI: 0.6, 8.7) and ear infection (OR=2.2; 95% CI: 1.0, 5.0). Reported concerns about outdoor sources of smoke were also associated with slightly, but non-significantly, elevated prevalence of reported pneumonia (OR=1.8; 95% CI: 0.6, 5.6) and bronchitis (OR=1.8; 95% CI: 0.6, 4.8) among children aged <5 years. Adult residents were also asked to comment on changes to the indoor environment that they would make to improve indoor air quality. Region 1 homes in which adults reported a desired change of improving air ventilation or purification were more likely to include child residents with reported pneumonia (OR=1.9; 95% CI: 0.7, 5.3) and throat infection (OR=2.2; 95% CI: 0.9, 5.5) (Table III).

Among children aged 5 to 17 years in Region 1 (Table IV), associations between heating type and reported respiratory infections generally demonstrated a similar (but weaker) pattern than described for children aged <5 years. For example, ORs for pneumonia and bronchitis and wood stove residence relative to fuel oil only heating were 1.5 (95% CI: 0.6, 4.0) and 1.5 (95% CI: 0.7, 3.3) compared to 2.1 (95% CI: 0.6, 7.2) and 2.0 (95% CI: 0.7, 6.3) as presented in Table III. For children aged 5 to 17 years, household smoking was associated with reported flu (OR=1.8; 95% CI: 1.1, 3.0). Only reporting of cold demonstrated a positive association with respect to concern with outdoor sources of smoke (OR=2.0; 95% CI: 0.8, 4.5). In addition, Region 1 homes in which adults reported concerns with indoor mould were more likely to include older children with reported flu (OR=2.0; 95% CI: 1.1, 3.7) or ear infection (OR=1.8; 95% CI: 1.2, 2.7). A desired change of air ventilation or purification was more likely to include child residents (aged 5 to 17 years) with reported pneumonia (OR=2.6; 95% CI: 1.3, 5.5) and flu (OR=2.2; 95% CI: 1.2, 3.9).

Table V presents risk estimates for home characteristics and reported asthma or respiratory symptoms (wheezing, dry cough, sinus congestion, watery or itchy eyes and productive cough) for all Region 1 children aged <18 years. Wheezing was the strongest symptom associated with household smoking (OR=2.1; 95% CI: 1.1, 4.0). Reported wheezing was also associated with residing in an older home (OR=2.0; 95% CI: 1.0, 3.8) but not with type of home heating. Homes with asthmatic children were more likely to have wood stove only heating relative to fuel oil only heating (OR=1.8; 95% CI: 0.6, 5.2), but this observation does not exclude the null. Wood stove only heating also showed a non-significant positive association with reported congestion (OR=2.0; 95% CI: 1.0, 3.9) and irritated eyes (OR=2.0; 95% CI: 0.8, 5.0). These associations were similar when household smoking was included in analyses (OR=1.9; 95% CI: 0.9, 3.8 for congestion and OR=2.0; 95% CI: 0.8, 4.9 for irritated eyes; data not shown). Although household crowding was not significantly linked to any measured respiratory outcome in either age group, it was significantly associated with a lower frequency of stuffy or runny nose. Among the concerns expressed by adult residents, presence of mould was associated with reporting of productive cough (OR=2.0; 95% CI: 1.1, 3.8). Concern with outdoor smoke was associated with dry cough at night (OR=2.3; 95% CI: 1.2, 4.3), congestion (OR=1.9; 95% CI: 1.0, 3.6) and productive cough (OR=1.9; 95% CI: 1.0, 3.8). A desired change of air ventilation or purification in households was associated with child wheezing (OR=2.1; 95% CI: 1.1, 4.2).

**Table V:** Odds ratios (OR, 95% CI) for asthma and respiratory symptoms and household variables among children aged <18 years in Region 1

	n	Asthma	Wheezing, whistling in chest	Dry cough at night	Stuffy, runny nose; sinus congestion	Watery, itchy or irritated eyes	Productive cough
Crowding							
< 2.5	262	1.00	1.00	1.00	1.00	1.00	1.00
≥ 2.5	205	0.6 (0.3, 1.4)	1.2 (0.7, 2.3)	0.9 (0.5, 1.6)	0.5 (0.3, 0.9)*	0.7 (0.4, 1.4)	0.8 (0.4, 1.5)
Heating^a^							
Fuel oil only	180	1.00	1.00	1.00	1.00	1.00	1.00
Fuel oil + woodstove	184	1.5 (0.6, 4.0)	1.0 (0.5, 2.1)	1.4 (0.7, 3.5)	1.4 (0.8, 2.4)	1.3 (0.6, 2.8)	0.7 (0.4, 1.4)
Woodstove only	85	1.8 (0.6, 5.2)	0.8 (0.3, 2.0)	1.5 (0.7, 3.2)	2.0 (1.0, 3.9)	2.0 (0.8, 5.0)	0.9 (0.4, 2.1)
Household smoker							
No	244	1.00	1.00	1.00	1.00	1.00	1.00
Yes	226	0.8 (0.3, 1.7)	2.1 (1.1, 4.0)*	1.6 (1.0, 2.8)	1.4 (0.9, 2.3)	1.1 (0.5, 2.0)	1.3 (0.7, 2.4)
Age of house							
Built 1985 or later	261	1.00	1.00	1.00	1.00	1.00	1.00
Built before 1985	193	1.2 (0.5, 2.6)	2.0 (1.0, 3.8)	1.4 (0.8, 2.4)	1.1 (0.7, 1.9)	1.1 (0.6, 2.2)	1.3 (0.7, 2.4)
Presence of mould^b^							
No	335	1.00	1.00	1.00	1.00	1.00	1.00
Yes	138	1.0 (0.5, 2.4)	1.1 (0.6, 2.3)	0.6 (0.3, 1.2)	1.1 (0.6, 2.0)	1.3 (0.6, 2.5)	2.0 (1.1, 3.8)
Concern of outdoor smoke^b^							
No	386	1.00	1.00	1.00	1.00	1.00	1.00
Yes	87	1.5 (0.6, 3.7)	0.9 (0.5, 1.7)	2.3 (1.2, 4.3)*	1.9 (1.0, 3.6)	1.6 (0.7, 3.5)	1.9 (1.0, 3.8)
Change – Ventilation/purification^b^							
No	353	1.00	1.00	1.00	1.00	1.00	1.00
Yes	120	0.9 (0.4, 2.0)	2.1 (1.1, 4.2)*	1.5 (0.8, 2.8)	1.6 (0.9, 2.7)	1.5 (0.7, 3.1)	1.7 (0.9, 3.3)

n=number of children; OR=odds ratio; CI=confidence interval.

a Does not include 8 children in homes with other type of heating.

b These non-mutually exclusive categories are based on open-ended comments by participants.

* p<0.05.

## Discussion

### Comparison of LRTI results to other studies

Regional variability in respiratory morbidity was identified in the 2 regions that participated in this study. Specifically, pneumonia prevalence was significantly (p<.05) higher among Region 1 children of both age groups compared to Region 2 children. Among children aged <5 years, approximately 20% of Region 1 children in this age group had bronchitis or pneumonia or both during the past year. Our findings are comparable to those findings presented in a previous study (13), where annual outpatient rates for LRTI among AI/AN children aged <5 years were reported as 250 per 1,000 children. Notably, the rates for AI/AN children were approximately 2-fold higher when compared to the annual rates for US children.

### Asthma prevalence in rural Alaska

As determined by the respiratory health questionnaires, reported asthma was slightly higher among children in Region 2 (9.3%) compared to Region 1 (7.6%). Although the reported prevalence of asthma determined in this study was lower than the national average (9.5% of US children currently have asthma), asthma-related symptoms were common among non-asthmatic children in both Region 1 and Region 2. Among these symptoms, children in Region 1 were significantly more likely to experience wheezing without the presence of a cold. In addition, children with reported asthma in Region 1 were also less likely to be currently using asthma medication, with only 30.6% of asthmatic children reportedly using their medication (compared to 75% in Region 2). These results may indicate a need for improved asthma screening in these regions, as well as improved patient education regarding the use of inhaled corticosteroids for the treatment of current asthma.

### Air quality questionnaire results and respiratory health

Over a 2-year period, our team administered 328 air quality questionnaires within 7 communities in rural Alaska. A full discussion of the air quality survey results is summarized in Ware et al. (27). Although a few studies have examined indoor air quality parameters in similar environments (29–31), little information exists regarding indoor air pollution and its impact on childhood respiratory health in Alaska Native communities.

When investigating the relationships between the air quality questionnaires and the results of the respiratory health questionnaires in Region 1, we were able to uncover important relationships between the 2 data sets. This was especially true for community concerns about wood smoke (indoor and ambient) and indoor mould. There was suggestive evidence that living in homes exclusively heated with wood was associated with increased prevalence of pneumonia, bronchitis, asthma, upper respiratory infections, congestion and irritated eyes among children. Concern about outdoor sources of smoke was also associated with elevated, but non-significant, prevalence estimates for LRTI among children aged <5 years. Concern with indoor mould was associated with increased ORs for nearly all reported respiratory infections, although these results were generally not significant. This is an important finding, as mould was the top indoor air quality concern reported in this region (27).

### Home characteristics and respiratory health

In rural Alaska, unfavourable weather conditions can lead to more time spent indoors. Other factors such as smoking, home age, wood stove use and house crowding have the potential for increased concentrations of indoor air contaminants and corresponding respiratory morbidity among children (26, 32). For all Region 1 children, smoking was significantly associated with reported wheezing. These findings are similar to those reported in Noonan et al. (33), where the risk for wheezing and other asthma-related symptoms was more than double in households with 1 or more smokers (33). Contrary to other studies (34–36), household smoking was not strongly associated with any of the reported respiratory infections among children aged <5 years and was only associated with reported flu for children aged 5 to 17 years. These findings are potentially explained by the low percentage of smokers who smoke indoors in Region 1. Despite high smoking rates in the region, previous studies have shown that 2% or less of smokers report smoking inside the home (37).

Statistical analyses evaluating the age of home as a predictor of respiratory disease indicate that older homes were associated with lower reported frequency for some respiratory conditions. By contrast, living in an older home was also associated with increased prevalence of wheezing among all children. The ventilation of older homes could contribute to these findings, but our study was not designed to assess the air infiltration efficiencies of these homes. Additional objective measures would be required to more fully evaluate these factors and assess the potential for a differential impact of this factor on respiratory infections versus asthma-related symptoms such as wheezing.

Contrary to our *a priori* assumption, household crowding was not significantly linked to any of the respiratory infections in either age group. Surprisingly, crowding was significantly associated with a lower frequency of stuffy or runny nose. It is possible that this was a spurious finding, but supporting evidence is provided by the protective, but not statistically significant, association between crowding and cold among children aged <5 years (OR=0.4; 95% CI: 0.4, 1.3). Stuffy or runny nose is a non-specific symptom. Without knowing the aetiology of these reported symptoms, it would be difficult to speculate on what might drive the observed inverse association in this population.

### Lifetime hospitalization visits among children

It has been estimated that the cost of RSV hospitalization in the YK Delta of Alaska was approximately $1,034 per child compared to $27 in the continental US (29). These findings can be explained partially by the elevated costs for healthcare utilization, including travel to a regional centre often required for secondary or tertiary care. In this study, survey results revealed high prevalence of hospitalization among children residing in these rural study areas. Across both regions, approximately 21% of children aged 5 to 17 years reported a lifetime hospitalization due to respiratory disease. Among children aged <5 years, nearly 25% in Region 1 had been hospitalized, compared to 15% in Region 2. These results are consistent with a previous study in Region 1, where 384 LRTI hospitalizations per 1,000 infants aged <1 year were reported in 1999–2000 (38).

### Study limitations

Although this study yielded important information regarding respiratory health among children in rural areas of Alaska, several limitations to this study should be noted. One limitation is the small population size in our study. In total, respiratory health data were collected for 561 children within 328 homes (475 children in Region 1 and 86 children in Region 2). Although the study population included a large percentage of children in these communities (approximately 70% of households in both regions and 85% of children in Region 1), the small sample size limited our ability to identify factors associated with respiratory health in children aged <1 year, an age group that is particularly susceptible to respiratory infections.

We cannot rule out the possibility that homes with wood stoves may also have more mould or dust and these characteristics could be driving the associations observed. The lack of plumbed water in some residences may also be an important confounder (18). Consistently elevated prevalence estimates were also not observed across all reported infections (e.g. reported flu was not associated with wood stove only homes). In addition, with the large number of statistical tests performed as part of this study, we would expect to observe significant associations by chance alone. As a result, significant findings must be viewed taking into consideration the multiple testing burden.

This is also a cross-sectional study based on self-reported information and, as a result, we are limited in being able to infer causal links and to assess with certainty the directionality of some observed relationships. Survey respondents may have been unable to correctly distinguish between influenza, cold, and other infections experienced by their children. Health outcomes misclassification is a noted limitation, but it is unlikely that such information error is differentially biased with respect to home characteristics. A related but potentially more substantial concern with respect to internal validity is the possibility of general reporting bias. This could be particularly problematic when evaluating the associations between resident-reported child's respiratory health and resident-reported subjective concerns of indoor and outdoor air quality. For example, we observed a statistically significant association between concern of outdoor air quality and dry cough at night, and associations between this concern and upper respiratory symptoms were of borderline significance. It is possible that these associations reflect over-reporting of children's symptoms by parents who were concerned about outdoor air quality. Because of the cross-sectional nature of this study, this bias could operate in the reverse direction as well with over-reporting of outdoor air concern among residents that had children with respiratory symptoms. However, it is difficult to make assumptions regarding this potential source of reporting bias as elevated ORs were not observed for all, or most, of the respiratory conditions and symptoms with respect to concern of outdoor air pollution.

## Conclusions

Although overall sample sizes were small and statistical significance generally was lacking, the results from this study indicate that there may be preventable air pollution exposures (including wood smoke and mould) leading to childhood respiratory disparities in these rural areas. In this study, a high prevalence of LRTI was observed among children in Region 1, and nearly 1 in 5 children from both regions had been hospitalized during their lifetime for respiratory disease or trouble breathing. Although the prevalence of asthma reported in this study was lower than the national average, this may be an underestimate. Findings from the respiratory health questionnaires suggest that additional research is needed to assess the impact of asthma-related symptoms and the potential for undiagnosed asthma cases in these communities. Further research is needed to assess exposures to PM_2.5_ and mould, and their associated impacts on respiratory health, particularly among susceptible populations such as young children within these rural communities.
